# Effect of saffron supplementation on the glycemic outcomes in diabetes: a systematic review and meta-analysis

**DOI:** 10.3389/fnut.2024.1349006

**Published:** 2024-03-15

**Authors:** Jiaxin Liu, Yang Yang, Yun Qi

**Affiliations:** Department of Endocrinology, Tianyou Hospital, Wuhan University of Science and Technology, Wuhan, China

**Keywords:** diabetes mellitus, glycemic outcomes, saffron, systematic review, meta-analysis

## Abstract

**Aim:**

This meta-analysis was conducted to investigate the impact of saffron supplementation on the glycemic outcomes in patients with diabetes.

**Methods:**

Eight electronic databases were systematically searched from inception to March 31, 2023. RCTs of patients with diabetes receiving saffron compared with placebo which reported glycemic control outcomes were identified. WMD and 95% CIs were pooled using fixed-effects or random-effects models, depending on the significance of heterogeneity.

**Results:**

Out of the 837 citations screened, ten RCTs were included in the systematic review and meta-analysis. A total of 562 participants were enrolled, with 292 assigned to the intervention group and 270 to the control group. Saffron was administered at a dose of 5 mg/day to 1 g/day. Compared with placebo, saffron supplementation significantly reduced FPG (WMD = −8.42 mg/dL; 95% CI: −13.37, −3.47; *p* = 0.001) and HbA1c (WMD = −0.22%; 95% CI: −0.33, −0.10; *p* < 0.001). However, there was no significant effect on insulin levels, QUICKI and HOMA-IR.

**Conclusion:**

Saffron is effective for patients with diabetes in terms of FPG and HbA1c, therefore, it appears to be a promising adjuvant for the glycemic control of DM. However, the overall methodological quality of the identified studies is heterogeneous, limiting the interpretation of the benefit of saffron in diabetes. More long-term follow-up, well-designed and large-scale clinical trials are warranted to draw definitive conclusions.

**Systematic review registration:**

The protocol of review was registered in International Prospective Register of Systematic Reviews (PROSPERO) (ID: CRD42023426353).

## 1 Introduction

Diabetes mellitus (DM) is a heterogeneous metabolic disorder typified by hyperglycemia, due to inadequate insulin secretion, impaired insulin effect, or both (1). The International Diabetes Federation (IDF) estimates that in 2021, the global diabetes prevalence will be 10.5% (536.6 million), which is predicted to be 12.2% (783.2 million) in 2045, leading to the increasing burden of diabetes worldwide (2). DM has been linked to the development of microvascular complications involving eyes, kidneys and nerves, as well as cardiovascular disease (CVD), abnormal pulmonary function, metabolic liver damage and increased risk of carcinogenesis (3). Consequently, there is a continuous demand for healthcare services, substantial costs to manage the disease and effective interventions to reduce incidence (4). Balanced diets, insulin therapy, regular exercise and pharmacotherapy are available to control diabetes. Nowadays, medicinal plants are also recommended as alternative or complementary therapy to increase insulin secretion and enhance insulin sensitivity (5, 6).

Saffron, the dried stigma of *Crocus sativus* L., is a perennial bulb of the Iridaceae family (7). In Iran, it is the most expensive spice, known as “Red Gold” (8). Compounds and ingredients consisting of saffron stigmas include crocin, picrocrocin, crocetin and safranal, with crocin responsible for the reddish colour of saffron (9). Based on the results of studies in animals and clinical trials, saffron and crocin exert significant pharmacological properties, such as hypoglycemic (10), hypolipidemic (11), antioxidant (12), anti-inflammatory (13), anticarcinogenic (14), neuroprotective (15), anti-depressive (16), and cardioprotective (17) activities, so it may have beneficial effects on diabetes, atherosclerosis, cancer, neurological disorders, depression and cardiovascular disease. Attributing to anti-inflammatory and antioxidant activities, saffron is considered to ameliorate metabolic disorders (18). Indeed, over the years, a variety of preclinical evidence and preliminary studies as well as clinical trials indicate that saffron and its constituents have antidiabetic effects. In an *in vitro* study investigating Moroccan and Italian saffron extracts, both extracts had a powerful antioxidant activity through the inhibition of 2,2-diphenyl-1-picrylhydrazyl (DPPH). The antidiabetic activity was evaluated by using alpha-amylase and alpha-glucosidase inhibition assay, suggesting that the compounds possessed hypoglycemic effects. Additionally, the disc diffusion method demonstrated that both extracts were effective against bacteria (19). Another experiment on 40 diabetic rats with an average age of 4 weeks was designed to evaluate the impacts of saffron petals and damask rose petals on inflammatory factors, fasting plasma glucose (FPG), hemoglobin A1c (HbA1c) and lipid profile. In the saffron petal group, insulin-like growth factor 1 (IGF-1), high-sensitivity C-reactive protein (hs-CRP), HbA1c, triglyceride was increased along with a decrease in FPG, which taken together reflected the beneficial effects of saffron on improving the status of biochemical markers (20). Although previous meta-analyses have reported the effect of saffron on glycemic parameters, results are inconsistent and none of them has focused on the whole DM population (21–26). Due to the lack of comprehensive meta-analytic evaluation of relevant randomized clinical trials (RCTs) published to date, we conducted a systematic review and meta-analysis to determine the effect of saffron supplementation on glycemic indices in patients with diabetes.

## 2 Materials and methods

This review was in accordance with the Preferred Reporting Items for Systematic Review and Meta-Analyses (PRISMA) statement (27). The protocol of review was registered in International Prospective Register of Systematic Reviews (PROSPERO) (ID: CRD42023426353).

### 2.1 Search strategy

A comprehensive search in PubMed, Web of Science (WOS), Embase, Cochrane Central Register of Controlled Trials (CENTRAL), Chinese BioMedical Literature Database (CBM), National Knowledge Infrastructure (CNKI), Wangfang Medicine Online (WANFANG), China Science and Technology Journal Database (VIP) was performed from reception to March 31, 2023, using MeSH (Medical Subject Headings) terms relating to DM and crocus. The keywords were (“Diabetes Mellitus” OR “Diabetes” OR “Diabetic” OR “DM”) AND (“Crocus” OR “Saffron” OR “Saffrons” OR “*Crocus sativus*” OR “Saffron Crocus” OR “Crocus, Saffron”). Furthermore, ClinicalTrials.gov and International Clinical Trials Registry Platform (ICTRP) were manually searched for the identification of potentially eligible studies. The detailed search strategy is given in Supplementary Table S1.

### 2.2 Eligibility criteria

The Patient, Intervention, Comparison, Outcome and Study design (PICOS) model (28) was applied to formulate the inclusion criteria as follows: (1) patients were diagnosed with DM, regardless of age, gender, race and diabetic complications; (2) the intervention group received saffron or its ingredients, without other unconventional interventions; (3) the control group received placebo; (4) at least one of the following outcomes was reported: FPG, HbA1c, insulin levels, quantitative insulin sensitivity check index (QUICKI), and homeostatic model assessment of insulin resistance (HOMA-IR); (5) RCTs without language limitations. The exclusion criteria were: (1) animal and *in vitro* studies; (2) reviews, meta-analyses, case reports, editorials, letters and notes; (3) lack of outcomes of interest; (4) data could not be extracted.

### 2.3 Study selection

Endnote Version X9 software was utilized to select and manage the relevant citations. After deduplication, two authors independently screened the headings and abstracts to eliminate irrelevant ones according to the predefined eligibility criteria. Subsequently, full-text publications were screened to verify eligibility. All eligible studies were included without language restrictions. Differences were settled by negotiation and consensus.

### 2.4 Data extraction

A standardized Microsoft Excel data extraction form was previously designed. Two authors independently extracted data relating to baseline characteristics of included trials, which consisted of: the first author, year of publication, registration number, study design, population, study arm, intervention duration, sample size, age, gender, body weight, body mass index (BMI), diabetes history, and outcomes. Baseline data were presented as mean and standard deviation (SD). If an included research expressed the data as the standard error of mean (SEM), we transformed it into SD. Discrepancies were settled by discussion and consensus.

### 2.5 Quality assessment

Two authors assessed the methodology quality according to the Cochrane Risk of Bias Tool for RCTs (29). This quality evaluation addressed aspects including random sequence generation (selection bias), allocation concealment (selection bias), blinding of participants and personnel (performance bias), blinding of outcome assessors (detection bias), incomplete outcome data (attrition bias), selective reporting (reporting bias) and other bias. Each domain was rated as “low,” “high,” or “unclear” risk of bias. The bias risk plots were generated with RevMan Version 5.3 software. To rigorously reduce potential biases, the quality evaluation of the included research was strictly carried out in accordance with the methods and principles of evidence-based medicine. Furthermore, evaluators contacted the authors to obtain complete data when there were unclear descriptions of research data in the included papers. If it could not be solved, we would exclude these literature. Additionally, sensitivity analyses were used to investigate whether different included studies had an impact on the results of meta-analysis. If the conclusions of the meta-analysis were reversed after leaving out one study, we should be alert whether there was a bias. Disagreements were resolved through appropriate discussion.

### 2.6 Statistical analysis

Statistics were analyzed using Stata Version 15.0 software. Glycemic control outcomes were measured by FPG, HbA1c, insulin levels, QUICKI and HOMA-IR. The variation in mean and SD values from baseline to final in the intervention and control arms was documented using Microsoft Excel in a tabular form. If the data were not described in the form of mean and SD, we calculated SD from SEM, 95% confidence intervals (95% CIs), *t*-value, *p*-value of *t*-test that relate to the differences between means. In case none of the above statistics was available from the trial report, we estimated the missing SD using a value, correlation coefficient (Corr = 0.5) as well as the baseline and final data according to the Cochrane Handbook (30). In the meta-analysis, differences between continuous outcome variables in two groups were determined using weighted mean difference (WMD), with 95% CIs when the unit of outcome measurements was consistent. Heterogeneity between the enrolled trials was evaluated using Cochrane’s *Q* test (Chi-square test) and *I*^2^ statistics, with a statistical significance set at a *p*-value of Cochrane’s *Q* test <0.10 or an *I* > 50%. To indicate the grade of heterogeneity, *I*^2^-values of 25, 50, and 75% were regarded as low, moderate, and high heterogeneity, respectively (31). Subgroup analysis and sensitivity analysis were applied to explore heterogeneity across enrolled studies, whereas a random-effects model was employed because of concerns about the magnitude of the heterogeneity that could not be addressed using the strategies described above. Subgroup analyses were conducted based on various study designs (single-blinded vs. double-blinded vs. triple-blinded), patients [type 2 diabetes mellitus (T2DM) vs. others], types (saffron vs. crocin), forms (tablet vs. capsule vs. others), doses (≤30 mg/day vs. >30 mg/day), and durations (<12 weeks vs. ≥12 weeks) of intervention. Furthermore, sensitivity analyses were performed using the leave-one-out method to test the robustness on the pooled effect. Publication bias was assessed by funnel plots when no less than ten included studies reported corresponding outcomes. Egger’s tests were further employed to test potential publication bias, when appropriate (32). Statistical significance was determined at a *p*-value of 0.05.

## 3 Results

### 3.1 Search results

**Figure 1:**
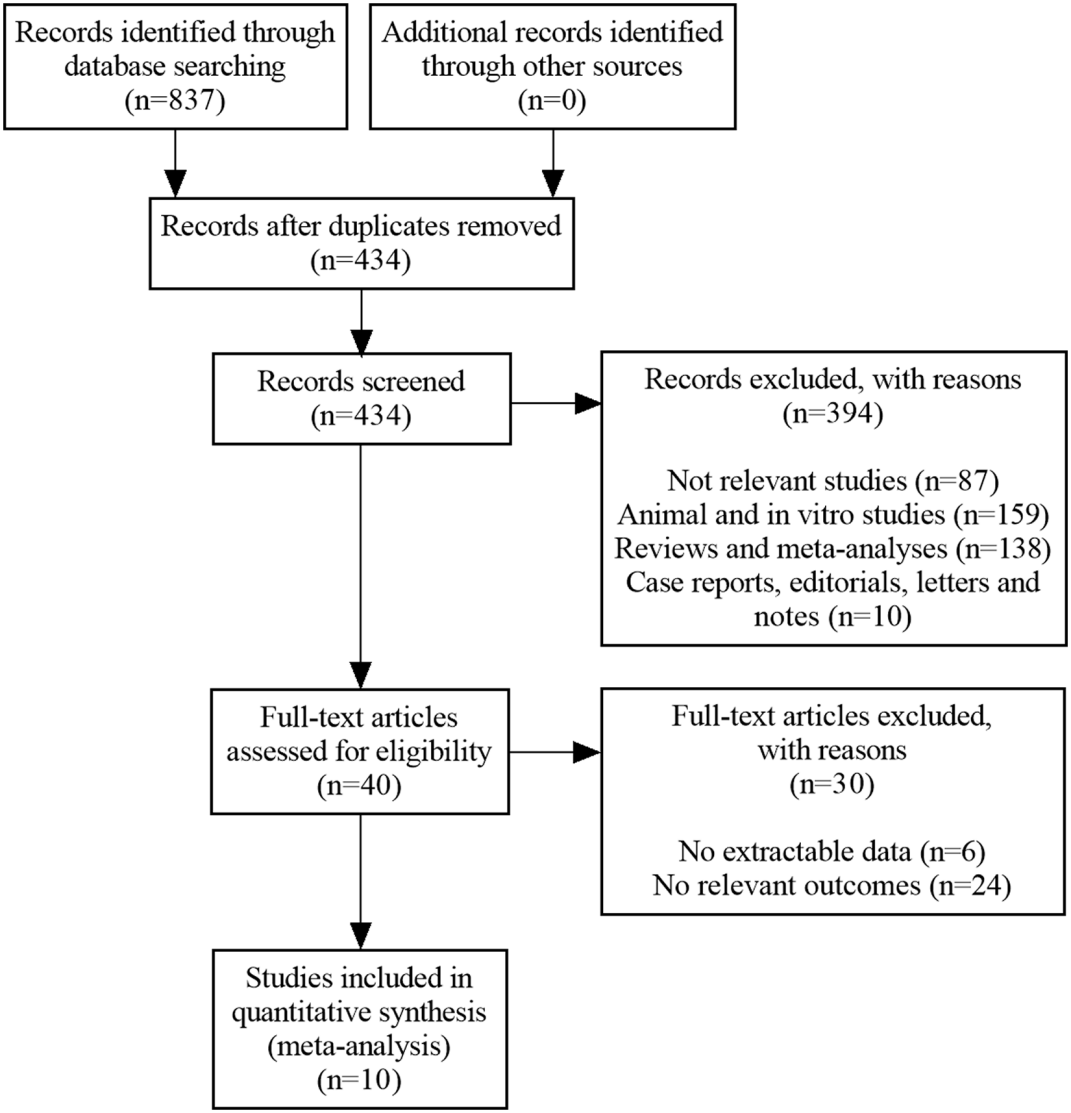
PRISMA flow diagram of study selection.

The literature search yielded a total of 837 citations (122 from PubMed, 222 from WOS, 240 from Embase, 72 from CENTRAL, 38 from CBM, 37 from CNKI, 65 from WANFANG, 41 from VIP and 0 from others). After the deletion of duplicate records, 434 relevant references remained. Subsequently, 394 were eliminated by screening the titles and abstracts. Afterwards, 40 papers were downloaded and identified in the phase of full-text screening, of which these were rejected on the grounds that they had: no extractable data (*n* = 6), or no relevant outcomes (*n* = 24). Finally, ten RCTs that met our eligibility criteria were enrolled (33–42). Figure 1 illustrates a PRISMA flow chart for the selection procedure.

### 3.2 Study characteristics

The detailed baseline characteristics of the ten RCTs are presented in Table 1. All papers were published in 2014 or later. Of those studies, nine registered the protocol in ICTRP, while one registered in ClinicalTrials.gov. All studies were designed in parallel groups. Three trials were triple-blinded, six were double-blinded, and one was single-blinded. Included studies enrolled subjects with T2DM, prediabetes, diabetic nephropathy and diabetic maculopathy. All of the included trials were conducted in Iran. Six studies evaluated saffron, three focused on crocin, and one compared saffron as well as crocin with placebo. Intervention doses ranged from 5 mg/day to 1 g/day. There were various forms of intervention among the trials, of which five used tablets, three were given capsules, one consumed black tea, and one received pills without mentioning the specific form. The duration of intervention also varied across studies: 8 weeks in four trials, and 12 weeks (3 months) in six trials. A total of 562 participants were enrolled, with 292 individuals assigned to the intervention group and 270 to the control group. In these studies, the average baseline age ranged from 50.57 to 63.86 years, with males accounting for 12 to 65%. The mean BMI varied from 23.84 to 31.2 kg/m^2^, while the mean weight ranged from 63.10 to 84.6 kg. The duration of diabetes ranged from 4.67 to 19.51 years.

**Table 1:** Characteristics of included studies.

Study ID	Registration number	Study design	Population	Study arm	Duration of intervention	Sample size	Age (years)	Male/Female	BMI (kg/m^2^)	Weight (kg)	Diabetes duration (years)	Outcome
Azimi, 2014 (30)	IRCT201206185062N5	Randomized, single-blind, placebo-controlled, parallel	T2DM	1 g/day saffron +3 glasses of black tea	8 weeks	42	57.02 ± 6.48	16/26	28.86 ± 1.30	81.97 ± 6.48	NR	①②③
				3 glasses of black tea		39	53.64 ± 8.12	15/24	28.40 ± 1.25	78.74 ± 7.49	NR	
Behrouz, 2020 (31)	NCT04163757	Randomized, double-blind, placebo-controlled, parallel	T2DM	30 mg/day crocin tablet	12 weeks	25	57.08 ± 7.41	4/21	30.64 ± 4.79	77.08 ± 10.18	NR	①②③④⑤
				Placebo		25	59.86 ± 9.46	3/22	30.85 ± 3.19	74.18 ± 7.97	NR	
Ebrahimi, 2019 (32)	IRCT201510259472N9	Randomized, double-blind, placebo-controlled, parallel	T2DM	200 mg/day saffron tablet	12 weeks	40	55.2 ± 7.3	20/20	29.3 ± 4.9	75.3 ± 12.8	7.8 ± 5.4	①②③④⑤
				Placebo		40	53 ± 10.6	16/24	30.5 ± 4.7	80.3 ± 12.8	6.6 ± 6.1	
Jaafarinia, 2022 (33)	IRCT20190810044500N4	Randomized, triple-blind, placebo-controlled, parallel	Diabetic nephropathy	15 mg/day crocin tablet	3 months	21	63.86 ± 10.62	12/9	27.21 ± 3.86	NR	13.20 ± 3.27	①②
				Placebo		19	62.68 ± 9.84	11/8	27.26 ± 3.34	NR	11.1 ± 7.48	
Karimi-Nazari, 2019 (34)	IRCT20120913010826N19	Randomized, double-blind, placebo-controlled, parallel	Prediabetes	15 mg/day saffron pill	8 weeks	36	57.95 ± 8.12	13/23	29.35 ± 1.50	76.29 ± 3.46	NR	①②
				Placebo		39	57.9 ± 8.7	14/25	28.78 ± 2.02	74.51 ± 4.55	NR	
Milajerdi, 2018 (35)	IRCT2015082623776N1	Randomized, triple-blind, placebo-controlled, parallel	T2DM	30 mg/day saffron capsule	8 weeks	26	54.57 ± 6.96	6/20	23.84 ± 11.89	63.10 ± 31.64	NR	①②
				Placebo		26	55.42 ± 7.58	6/20	28.30 ± 3.24	66.34 ± 9.01	NR	
Moravej Aleali, 2019 (36)	IRCT2015110219739N1	Randomized, double-blind, placebo-controlled, parallel	T2DM	30 mg/day saffron capsule	3 months	32	53.5 ± 9.9	8/24	28.8 ± 4.0	NR	7.84 ± 7.02	①②③④⑤
				Placebo		32	52.4 ± 13	11/21	27.5 ± 4.2	NR	4.68 ± 4.78	
Sepahi, 2018 (37)	IRCT2015062113058N2	Randomized, double-blind, placebo-controlled, parallel	Diabetic maculopathy	5 mg/day crocin tablet	3 months	20	54.31 ± 6.6	6/14	NR	NR	18.06 ± 1.83	①②
				15 mg/day crocin tablet		20	56.09 ± 4.3	13/7	NR	NR	18.63 ± 1.58	
				Placebo		20	57.17 ± 2.9	10/10	NR	NR	19.51 ± 1.41	
Sepahi, 2022 (38)	IRCT2015101713058N3	Randomized, triple-blind, placebo-controlled, parallel	T2DM	30 mg/day crocin tablet	3 months	50	57.58 ± 1.0	22/28	NR	NR	8.81 ± 0.9	①②③⑤
				30 mg/day saffron tablet		50	57.16 ± 1.5	21/29	NR	NR	8.22 ± 0.9	
				Placebo		50	56.92 ± 1.9	25/25	NR	NR	8.46 ± 1.9	
Tajaddini, 2023 (39)	IRCT20090609002017N24	Randomized, double-blind, placebo-controlled, parallel	T2DM	100 mg/day saffron capsule	8 weeks	30	50.57 ± 9.88	15/15	30.0 ± 4.2	82.7 ± 11.3	6.30 ± 4.53	①②③⑤
				Placebo		30	51.83 ± 10.91	13/17	31.2 ± 4.6	84.6 ± 14.4	4.67 ± 4.35	

NR, not reported; T2DM, type 2 diabetes mellitus; BMI, body mass index. ① FPG, ② HbA1c, ③ insulin levels, ④ QUICKI, and ⑤ HOMA-IR. Data are expressed as mean ± SD.

### 3.3 Risk of bias assessment

**Figure 2:**
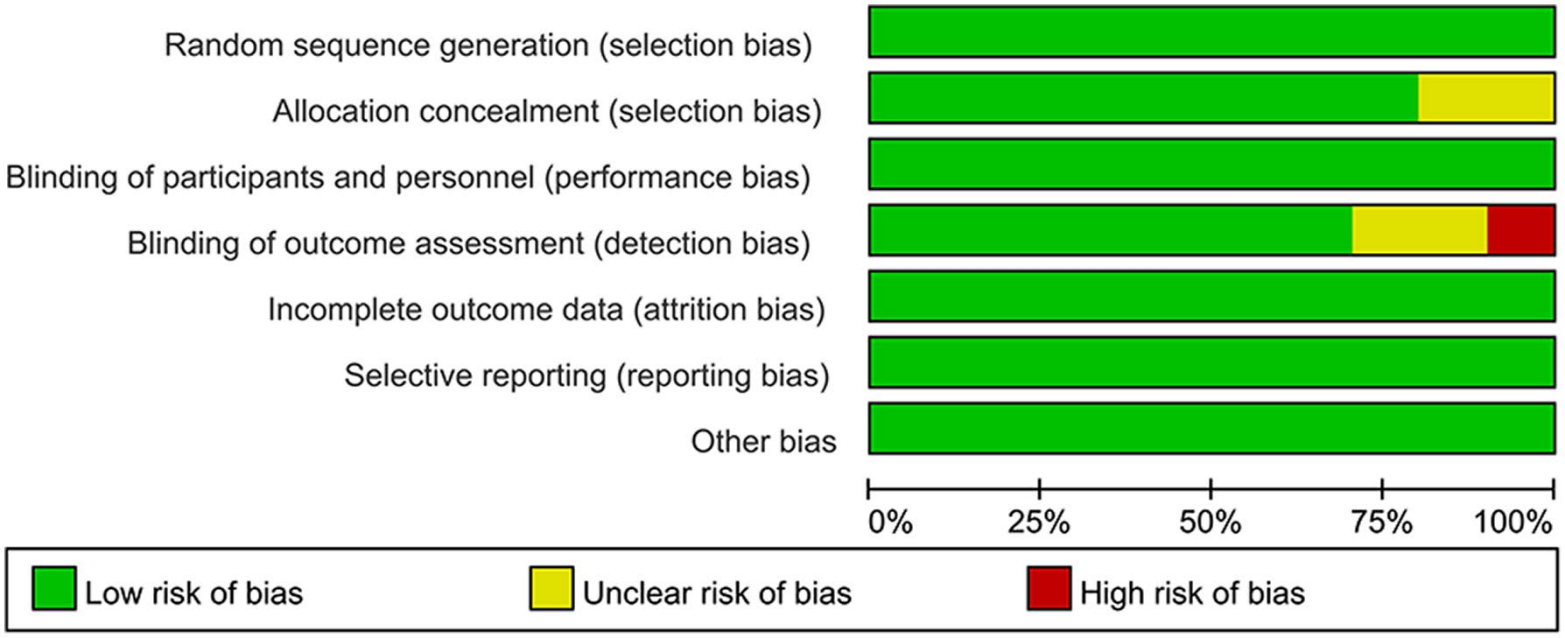
Risk of bias graph for included studies.

**Figure 3:**
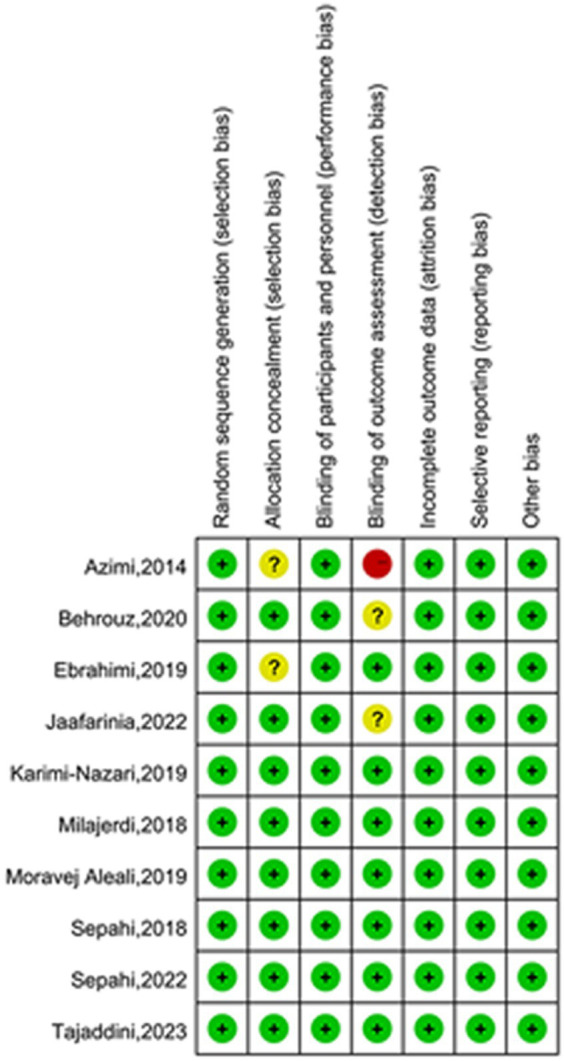
Risk of bias summary for included studies.

All ten included studies described methods for random sequence generation and blinding. Eight papers described details of allocation concealment, whereas the remaining two papers did not. The absence of appropriate blinding of outcome assessment in one article led to a high risk of bias in the domain of detection bias, and two articles were considered to have an unclear risk of bias due to failure of describing blinding of outcome assessors. Preselected outcomes were comprehensively reported in all of the studies. None reported selective outcomes. The quality evaluation graph and summary are provided in Figures 2, 3.

### 3.4 Pooled analysis

#### 3.4.1 Effect on FPG

**Figure 4:**
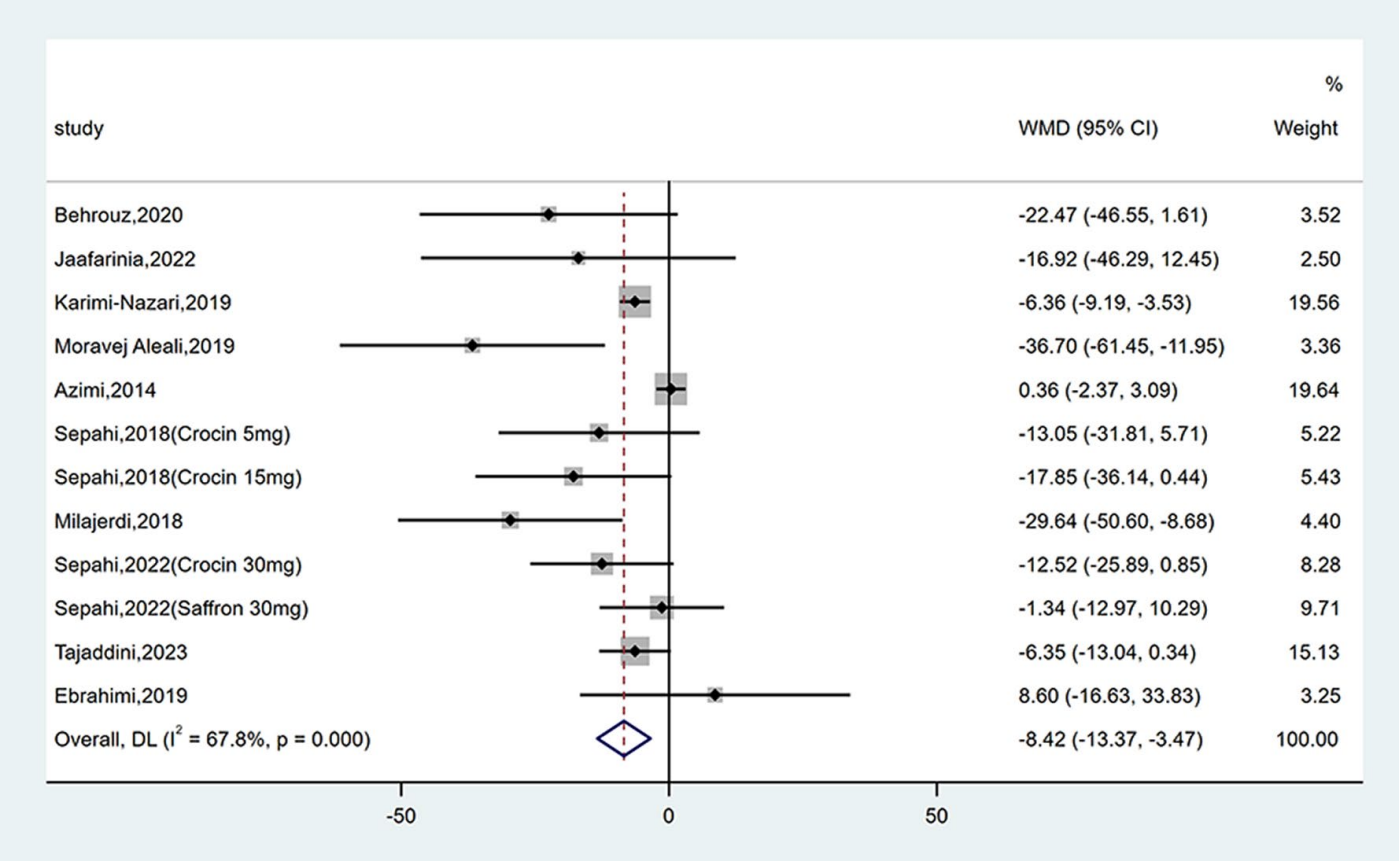
Forest plot of the effect of saffron supplementation on FPG.

Twelve studies reported data on FPG. As shown in Figure 4, saffron significantly reduced FPG levels in comparison with the control group (WMD = −8.42 mg/dL; 95% CI: −13.37, −3.47; *p* = 0.001). There was moderate heterogeneity (*I*^2^ = 67.8%, *p* < 0.001). Subgroup analyses were conducted according to different study designs, patients, durations, types, forms and doses of intervention (Table 2). Results revealed a greater reduction on FPG in subgroups of triple-blinded studies (WMD = −12.55 mg/dL; 95% CI: −24.45, −0.66; *p* = 0.039), patients diagnosed with T2DM (WMD = −9.05 mg/dL; 95% CI: −16.64, −1.46; *p* = 0.019), crocin supplementation (WMD = −15.30 mg/dL; 95% CI: −23.66, −6.93; *p* < 0.001), capsules intake (WMD = −21.77 mg/dL; 95% CI: −42.80, −0.74; *p* = 0.043). Additionally, there was a statically significant decrease in FPG when dose of intervention was ≤30 mg/day (WMD = −13.19 mg/dL; 95% CI: −19.99, −6.40; *p* < 0.001) and duration of intervention was ≥12 weeks (WMD = −12.39 mg/dL; 95% CI: −20.73, −4.06; *p* = 0.004).

**Table 2:** Summary of subgroup analyses of saffron on FPG, HbA1c, insulin levels, QUICKI, and HOMA-IR in diabetes.

Subgroup	Studies included	WMD	95% CI	*p* of pooling WMD	Heterogeneity		
					*I*^2^%	*p*	*p* between sub-group
FPG (mg/dL)							
Study design							0.002
Single-blinded	1	0.36	−2.37, −3.09	0.796	–	–	
Double-blinded	7	−9.57	−15.53, −3.60	**0.002**	43.2	0.103	
Triple-blinded	4	−12.55	−24.45, −0.66	**0.039**	48.7	0.119	
Type of diabetes							0.595
T2DM	8	−9.05	−16.64, −1.46	**0.019**	71.5	0.001	
Others	4	−6.86	−9.61, −4.11	**<0.001**	0.0	0.498	
Type of intervention							0.070
Saffron	7	−5.98	−11.62, −0.34	**0.038**	77.1	<0.001	
Crocin	5	−15.30	−23.66, −6.93	**<0.001**	0.0	0.958	
Form of intervention							0.150
Tablet	7	−9.41	−16.25, −2.57	**0.007**	5.6	0.384	
Capsule	3	−21.77	−42.80, −0.74	**0.043**	77.7	0.011	
Others	2	−2.99	−9.58, 3.60	0.374	91.1	0.001	
Dose of intervention (mg/day)							0.010
≤30	9	−13.19	−19.99, −6.40	**<0.001**	47.8	0.053	
>30	3	−1.62	−7.21, 3.97	0.571	47.6	0.148	
Duration of intervention (weeks)							0.189
<12	4	−5.56	−11.44, 0.32	0.064	83.6	<0.001	
≥12	8	−12.39	−20.73, −4.06	**0.004**	35.0	0.149	
HbA1c (%)							
Study design							0.001
Single-blinded	1	0.00	−0.10, 0.10	1.000	–	–	
Double-blinded	7	−0.21	−0.30, −0.12	**<0.001**	7.6	0.370	
Triple-blinded	4	−0.36	−0.55, −0.16	**<0.001**	0.0	0.819	
Type of diabetes							0.637
T2DM	8	−0.27	−0.48, −0.05	**0.014**	54.9	0.030	
Others	4	−0.21	−0.34, −0.07	**0.003**	24.5	0.264	
Type of intervention							0.113
Saffron	7	−0.15	−0.28, −0.03	**0.018**	57.5	0.028	
Crocin	5	−0.38	−0.62, −0.13	**0.002**	39.3	0.159	
Form of intervention							0.168
Tablet	7	−0.33	−0.49, −0.18	**<0.001**	9.9	0.353	
Capsule	3	−0.22	−0.67, 0.23	0.340	0.0	0.855	
Others	2	−0.10	−0.29, 0.09	0.291	91.6	0.001	
Dose of intervention (mg/day)							0.001
≤30	9	−0.26	−0.36, −0.15	**<0.001**	19.4	0.271	
>30	3	−0.01	−0.11, 0.08	0.817	0.0	0.530	
Duration of intervention (weeks)							0.042
<12	4	−0.10	−0.26, 0.07	0.242	74.9	0.008	
≥12	8	−0.32	−0.46, −0.18	**<0.001**	0.0	0.465	
Insulin levels (mU/l)							
Study design							0.004
Single-blinded	1	0.13	−0.07, 0.33	0.198	-	-	
Double-blinded	4	−0.59	−1.51, 0.34	0.213	68.1	0.025	
Triple-blinded	2	3.13	1.15, 5.10	**0.002**	3.4	0.309	
Type of intervention							0.783
Saffron	5	0.01	−0.28, 0.30	0.935	68.2	0.013	
Crocin	2	−0.86	−7.03, 5.32	0.786	90.4	0.001	
Form of intervention							0.243
Tablet	4	0.56	−1.97, 3.09	0.665	84.0	<0.001	
Capsule	2	−0.51	−1.25, 0.23	0.175	0.0	0.588	
Others	1	0.13	−0.07, 0.33	0.198	–	–	
Dose of intervention (mg/day)							0.849
≤30	3	0.43	−4.05, 4.92	0.850	83.4	<0.001	
>30	4	−0.00	−0.17, 0.17	0.968	52.0	0.125	
Duration of intervention (weeks)							
<12	2	−0.08	−0.66, 0.50	0.787	61.6	0.107	0.694
≥12	5	0.42	−2.02, 2.87	0.734	79.1	0.001	
HOMA-IR							
Study design							0.024
Double-blinded	4	−0.55	−1.19, 0.09	0.093	76.1	0.006	
Triple-blinded	2	1.04	−0.19, 2.26	0.097	48.4	0.164	
Type of intervention							0.498
Saffron	4	0.07	−0.51, 0.65	0.813	69.8	0.019	
Crocin	2	−0.82	−3.33, 1.69	0.521	89.6	0.002	
Form of intervention							0.673
Tablet	4	−0.02	−1.23, 1.19	0.970	84.9	<0.001	
Capsule	2	−0.46	−2.12, 1.19	0.582	8.9	0.295	
Dose of intervention (mg/day)							0.944
≤30	4	−0.27	−2.38, 1.84	0.801	85.7	<0.001	
>30	2	−0.19	−0.43, 0.04	0.100	10.4	0.291	
Duration of intervention (weeks)							0.801
<12	1	−0.28	−0.54, −0.02	**0.037**	–	–	
≥12	5	−0.12	−1.32, 1.08	0.841	81.0	<0.001	

The random-effects meta-analysis model (DerSimonian-Laird method) was used.

#### 3.4.2 Effect on HbA1c

**Figure 5:**
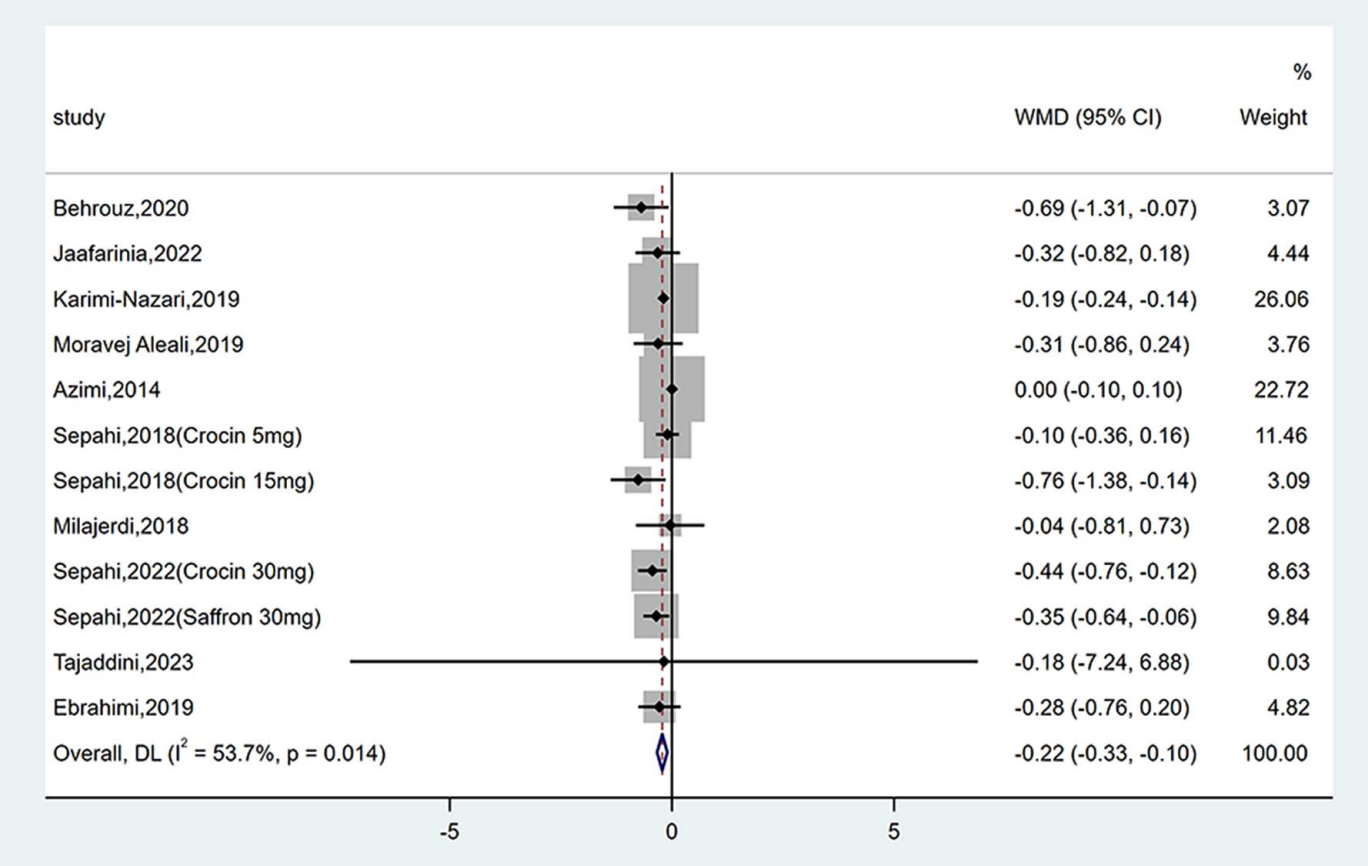
Forest plot of the effect of saffron supplementation on HbA1c.

Twelve studies reported data on HbA1c. As shown in Figure 5, saffron significantly reduced HbA1c levels compared with the control group (WMD = −0.22%; 95% CI: −0.33, −0.10; *p* < 0.001). The heterogeneity was moderate (*I*^2^ = 53.7%, *p* = 0.014). Subgroup analyses were performed by different study designs, patients, durations, types, forms, and doses of intervention (Table 2). Results revealed a greater reduction on FPG in subgroups of triple-blinded studies (WMD = −0.36%; 95% CI: −0.55, −0.16; *p* < 0.001), patients diagnosed with T2DM (WMD = −0.27%; 95% CI: −0.48, −0.05; *p* = 0.014), crocin supplementation (WMD = −0.38%; 95% CI: −0.62, −0.13; *p* = 0.002). In addition, there was a significant decrease on HbA1c when tablets were consumed (WMD = −0.33%; 95% CI: −0.49, −0.18; *p* < 0.001), dose of intervention was ≤30 mg/day (WMD = −0.26%; 95% CI: −0.36, −0.15; *p* < 0.001), and duration of intervention was ≥12 weeks (WMD = −0.32%; 95% CI: −0.46, −0.18; *p* < 0.001).

#### 3.4.3 Effect on insulin levels

**Figure 6:**
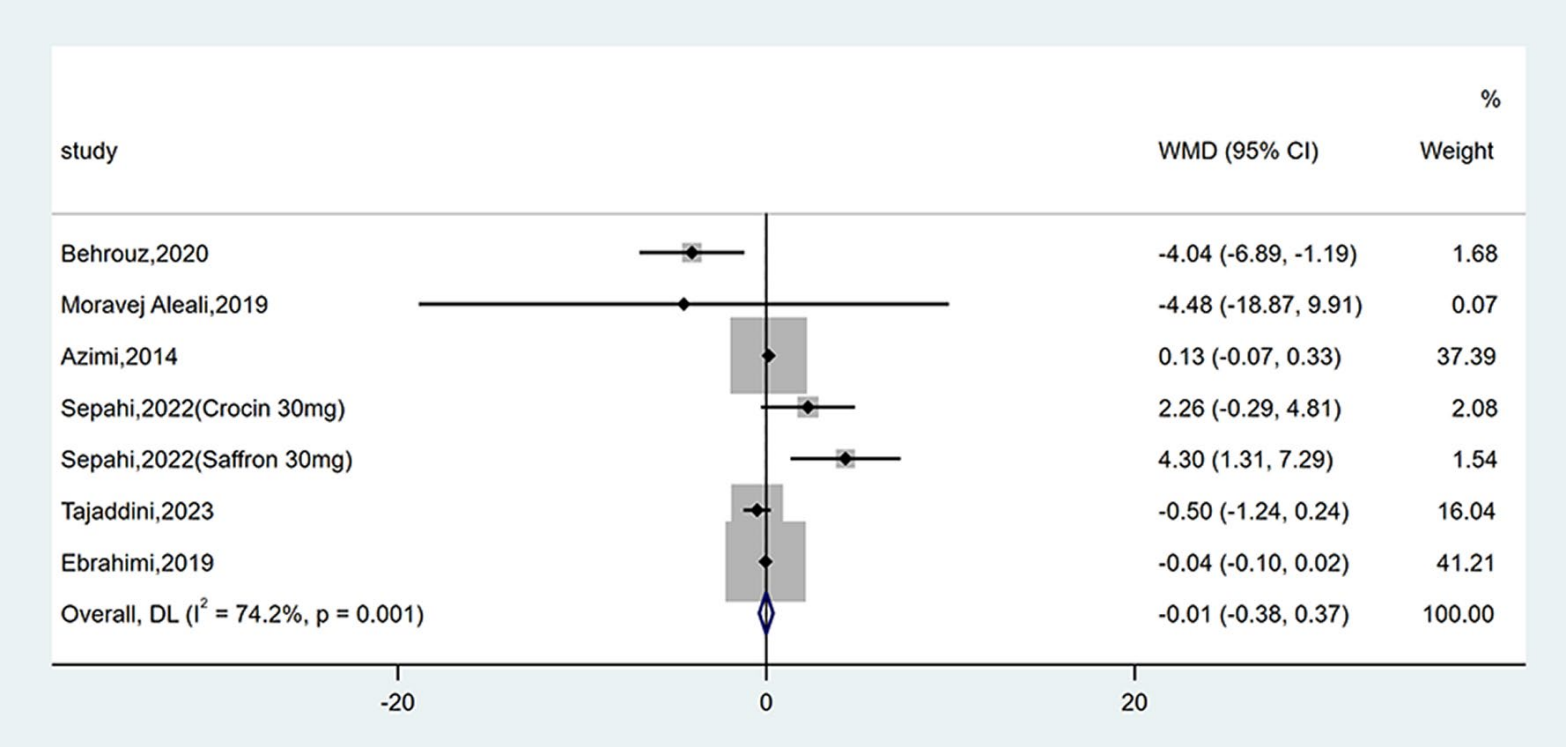
Forest plot of the effect of saffron supplementation on insulin levels.

Seven studies reported data on insulin levels. As shown in Figure 6, no statistical change was observed between groups (WMD = −0.01 mU/L; 95% CI: −0.38, 0.37; *p* = 0.975). There was moderate heterogeneity (*I*^2^ = 74.2%, *p* = 0.001). Subgroup analyses were conducted according to different study designs, types, forms, doses and durations of intervention (Table 2). Results revealed insulin levels had an increase in triple-blinded studies (WMD = 3.13 mU/L; 95% CI: 1.15, 5.10; *p* = 0.002), while subgroup analyses based on types, forms, doses and durations of intervention did not reveal significant change.

#### 3.4.4 Effect on QUICKI

**Figure 7:**
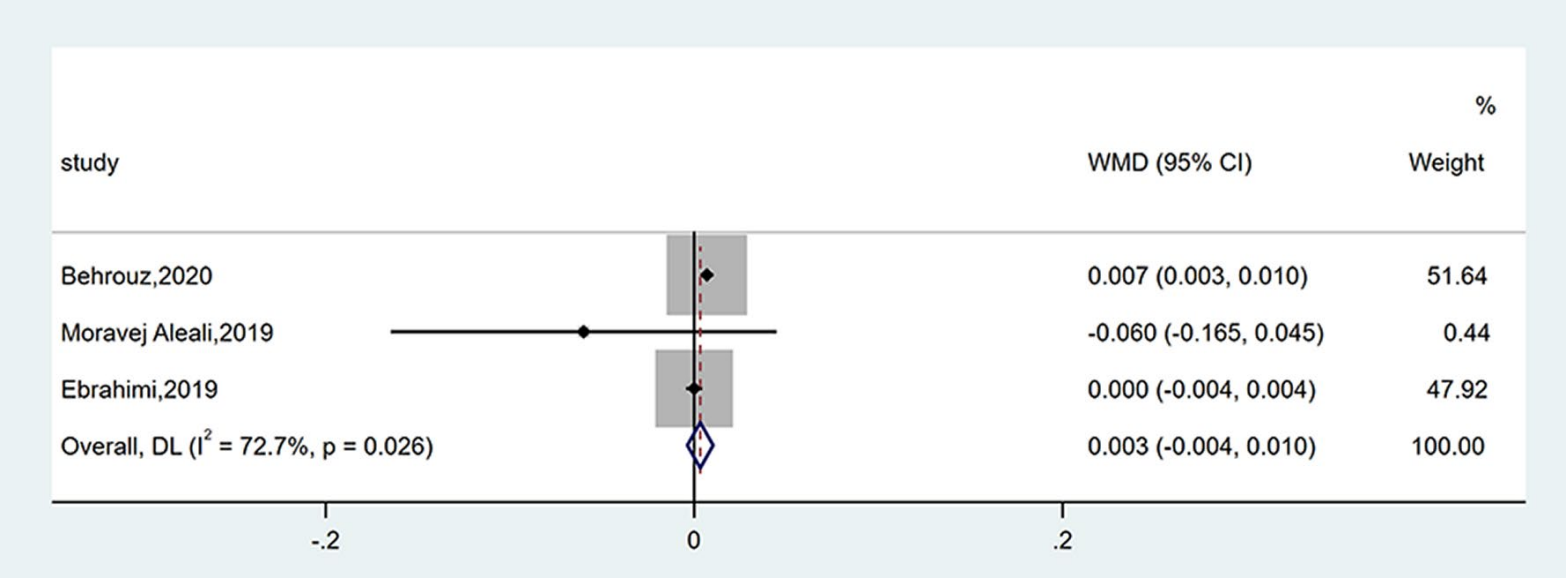
Forest plot of the effect of saffron supplementation on QUICKI.

Three studies reported data on QUICKI. There was no obvious variation between groups (WMD = 0.003; 95% CI: −0.004, 0.010; *p* = 0.355), as shown in Figure 7. The heterogeneity was moderate (*I*^2^ = 72.7%, *p* = 0.026). The number of enrolled trials was so limited that subgroup analysis was not carried out.

#### 3.4.5 Effect on HOMA-IR

**Figure 8:**
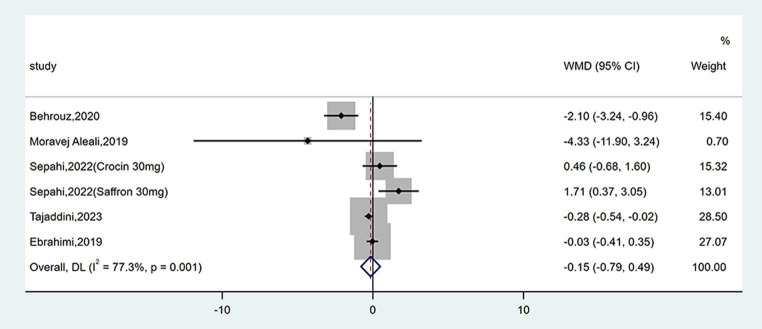
Forest plot of the effect of saffron supplementation on HOMA-IR.

Six studies reported data on HOMA-IR. No remarkable difference between groups was found (WMD = −0.15; 95% CI: −0.79, 0.49; *p* = 0.649), as shown in Figure 8. There was high heterogeneity (*I*^2^ = 77.3%, *p* = 0.001). Subgroup analyses were conducted according to different study designs, types, forms, doses and durations of intervention (Table 2). Results revealed that there was a significant decrease on HOMA-IR when duration of intervention was <12 weeks (WMD = −0.28; 95% CI: −0.54, −0.02; *p* = 0.037), while subgroup analyses based on study designs, types, forms and doses of intervention did not reveal a significant difference.

### 3.5 Sensitivity analysis

Sensitivity analysis was performed by excluding each included study in a sequential manner, without changing the significance or direction of the pooled effect. The leave-one-out sensitivity analyses indicated our findings were robust (Supplementary Figures S1–S5).

### 3.6 Publication bias

Funnel plots of FPG and HbA1c are illustrated in Supplementary Figures S6, S7. Egger’s test indicated no potential publication bias for HbA1c (*p* = 0.284), insulin levels (*p* = 0.828), QUICKI (*p* = 0.597) and HOMA-IR (*p* = 0.966), except for FPG (*p* = 0.036), which may arouse from poor quality of included studies, small sample size or moderate heterogeneity.

## 4 Discussion

In the present study involving ten RCTs, we evaluated the effect of saffron supplementation on the glycemic outcomes in patients with diabetes. We observed that saffron significantly reduced FPG and HbA1c levels compared to placebo, while the results did not show a significant effect on insulin levels, QUICKI and HOMA-IR following saffron supplementation.

In clinical practice, FPG is one of the criteria for the diagnosis of diabetes and it is also used to evaluate relatively short-term treatment compliance. HbA1c is an indicator of long-term glycemic control, because it provides information about glycemic status of the past 8–12 weeks (43). In our current study, saffron significantly reduced FPG and HbA1c levels, indicating its important role in glycemic control. People who depend on traditional antidiabetic drugs would benefit from it, for they could maintain better blood glucose control and HbA1c levels.

QUICKI emerges as a dependable and reproducible approach that accurately predicts insulin sensitivity, and it has a positive predictive power for the development of diabetes (44). HOMA-IR serves as an index for steady insulin resistance, effectively reflecting the reciprocal interaction between the liver and b-cell. It demonstrates commendable performance in estimating both insulin resistance and b-cell deficiency, aligning with the euglycemic clamp method, the gold standard of assessing b-cell function (45). In terms of insulin requirements, QUICKI and HOMA-IR values, we found no significant effects. This phenomenon demonstrated that the improvement of insulin resistance and sensitivity might not be the dominant therapeutic effect of saffron.

It has been demonstrated that saffron extract or crocin has the potential to improve glycemic profile in DM. A previous RCT reported that saffron supplementation significantly lowered FPG, HbA1c, insulin levels and HOMA-IR in obese men with T2DM (46). Another recent RCT revealed that 400 mg daily saffron supplementation in combination with 8 weeks aerobic training, could improve blood glucose, insulin levels and HOMA-IR in middle-aged overweight female patients with T2DM (47). The present meta-analysis revealed that saffron supplementation significantly ameliorated FPG and HbA1c levels, but we were unable to find any significant impact of saffron on insulin levels, QUICKI and HOMA-IR. The discrepancy between the results may be explained by different populations, doses, forms, and duration of intervention.

Previous animal studies have also confirmed the benefits of saffron and crocin on glycemic control. Mohajeri et al. found that the 20, 40, and 80 mg/kg of ethanolic extract of saffron induced distinct reduction of plasma glucose in alloxan-diabetic rats (48). Rajaei et al. reported that intraperitoneal pretreatment with 60 mg/kg of crocin notably reduced blood glucose levels in streptozotocin-induced diabetic rats (49). Ouahhoud et al. found that oral administration of *Crocus sativus* tepals, stigmas and leaves extracts markedly lowered blood glucose levels in the diabetic rats (50). In this regard, we found that saffron significantly lowered FPG and HbA1c levels. Specifically, the reduction was greater when doses of crocin ≤30 g/day were consumed, the supplementation duration was more than 12 weeks and in T2DM.

Our study revealed no significant effect on insulin levels, QUICKI and HOMA-IR following saffron supplementation. Recent studies in animals have explored the effect of saffron on insulin resistance. Shirali et al. reported that fasting insulin levels and HOMA-IR significantly decreased in the diabetic rats following the treatment with 50 and 100 mg/kg of crocin (51). Dehghan et al. found that after 6 weeks resistance exercise and 40 mg/kg of saffron treatment, serum glucose levels, HbA1c and HOMA-IR decreased but insulin levels were not significantly different (52). The discordance between the results of animal studies may arise from varying doses, forms and durations of administration.

It appears that the benefit of saffron on glycemic control in subjects with diabetes is related to attenuation of oxidative stress (53). Saffron aqueous extract improves oxidative stress by increasing the amounts of antioxidant enzymes such as catalase (CAT), glutathione peroxidase (GPx) and superoxide dismutase (SOD), decreasing malondialdehyde (MDA) (54). Crocin also plays a beneficial role in decreasing the mRNA expression of SOD, CAT, and GPx (55). In addition, saffron stimulates the translocation of glucose transporter 4 (GLUT-4) into the cell membrane via activating AMP-activated protein kinase (AMPK)/acetyl-CoA carboxylase (ACC) and mitogen-activated protein kinases (MAPKs) in skeletal muscle cells, thus enhancing glucose uptake and insulin sensitivity (56). Moreover, saffron alleviates diabetes by inhibiting inflammatory responses. It has been proposed that saffron and crocin can suppress and down-regulate mRNA of tumor necrosis factor-α (TNF-α) and interleukin-6 (IL-6) in abdominal aorta (57, 58), interleukin-17 (IL-17), high-sensitivity C-reactive protein (hs-CRP), TNF-α and nuclear factor-kappa B (NF-κB) in peripheral blood mononuclear cells (59). Crocin has been demonstrated to inhibit inflammatory reactions by lowering the level of interleukin-18 (IL-18), a potent proinflammatory cytokine that involves in the onset of diabetes nephropathy (60). Additionally, hypoglycemic potential of saffron stigma extract may be attributed to the regulation of insulin release and glucose metabolism. Through raising glucokinase (GK) expression and reducing glucose-6-phosphatase (G6Pase) expression, saffron can better improve gluconeogenesis (61).

The present study is the first meta-analysis of RCTs exploring the glycemic efficacy of saffron alone in DM patients. A previous meta-analysis investigated the effect of saffron supplementation on glycemic indices, but participants were not limited in DM (25). In our meta-analysis, we focused on patients with diabetes and included four additional recently published trials, increasing the credibility of our findings. In addition, most included RCTs were well designed as double-blinded or triple-blinded trials and possessed high quality, with the exception of one single-blinded trial.

There are some limitations to be recognized. First, the present meta-analysis includes a relatively limited number of RCTs with a small sample size and a short duration of intervention, especially in terms of insulin levels, QUICKI and HOMA-IR, which may have contributed to the lack of significant effect of saffron on the above parameters. Second, there was moderate to high heterogeneity across studies, possibly related to variations in study design, type of diabetes, dose, form, and duration of intervention. Third, all the trials in this meta-analysis were carried out in Iran, resulting in a limited representation of other countries. Also, due to only three trials reporting QUICKI data, we fail to perform subgroup analysis for this variable. Correspondingly, the findings require an interpretation with caution, and more RCTs with larger sample sizes and prolonged intervention durations are warranted to further explore the glycemic control, safety and tolerability of saffron in diabetic patients.

## 5 Conclusion

The findings support that saffron is able to reduce FPG and HbA1c levels, despite the fact that saffron does not appear to attenuate insulin resistance and sensitivity, it may have therapeutic potential as a promising adjuvant for glycemic control of DM. The significant heterogeneity among the included studies warrants more long-term follow-up, well-designed and large-scale clinical trials to elucidate the role of saffron in the treatment of DM.

## Data availability statement

The original contributions presented in the study are included in the article/Supplementary material, further inquiries can be directed to the corresponding authors.

## Author contributions

JL: Data curation, Formal analysis, Investigation, Methodology, Software, Validation, Visualization, Writing – original draft. YY: Conceptualization, Formal analysis, Funding acquisition, Supervision, Writing – review & editing, Methodology. YQ: Conceptualization, Formal analysis, Project administration, Resources, Writing – review & editing, Methodology.

## References

[1] Punthakee Z Goldenberg R Katz P. Definition, classification and diagnosis of diabetes, prediabetes and metabolic syndrome. Can J Diabetes. (2018) 42:S10–5. doi: 10.1016/j.jcjd.2017.10.003, PMID: 29650080

[2] Sun H Saeedi P Karuranga S Pinkepank M Ogurtsova K Duncan BB . IDF diabetes atlas: global, regional and country-level diabetes prevalence estimates for 2021 and projections for 2045. Diabetes Res Clin Pract. (2022) 183:109119. doi: 10.1016/j.diabres.2021.109119, PMID: 34879977 PMC11057359

[3] Mauricio D Alonso N Gratacòs M. Chronic diabetes complications: the need to move beyond classical concepts. Trends Endocrinol Metab. (2020) 31:287–95. doi: 10.1016/j.tem.2020.01.007, PMID: 32033865

[4] Gregg EW Zhuo X Cheng YJ Albright AL Narayan KMV Thompson TJ. Trends in lifetime risk and years of life lost due to diabetes in the USA, 1985-2011: a modelling study. Lancet Diabetes Endocrinol. (2014) 2:867–74. doi: 10.1016/S2213-8587(14)70161-5, PMID: 25128274

[5] Kooti W Farokhipour M Asadzadeh Z Ashtary-Larky D Asadi-Samani M. The role of medicinal plants in the treatment of diabetes: a systematic review. Electron Physician. (2016) 8:1832–42. doi: 10.19082/1832, PMID: 26955456 PMC4768936

[6] Eddouks M Bidi A El Bouhali B Hajji L Zeggwagh NA. Antidiabetic plants improving insulin sensitivity. J Pharm Pharmacol. (2014) 66:1197–214. doi: 10.1111/jphp.12243 24730446

[7] Melnyk JP Wang S Marcone MF. Chemical and biological properties of the world’s most expensive spice: saffron. Food Res Int. (2010) 43:1981–9. doi: 10.1016/j.foodres.2010.07.033

[8] Bathaie SZ Mousavi SZ. New applications and mechanisms of action of saffron and its important ingredients. Crit Rev Food Sci Nutr. (2010) 50:761–86. doi: 10.1080/10408390902773003, PMID: 20830635

[9] Tahereh F Saeed S. The effect of saffron (Crocus sativus L.) and its ingredients on the management of diabetes mellitus and dislipidemia. Afr J Pharm Pharmaco. (2014) 8:541–9. doi: 10.5897/AJPPX2013.0006

[10] Kianbakht S Hajiaghaee R. Anti-hyperglycemic effects of saffron and its active constituents, crocin and safranal, in alloxan-induced diabetic rats. J Medicinal Plants. (2011) 10:82–9.

[11] Arasteh A Aliyev A Khamnei S Delazar A Mesgari M Mehmannavaz Y. Effects of hydromethanolic extract of saffron (Crocus sativus) on serum glucose, insulin and cholesterol levels in healthy male rats. J Medicinal Plants Res. (2010) 4:397–402.

[12] Hasanpour M Ashrafi M Erjaee H Nazifi S. The effect of saffron aqueous extract on oxidative stress parameters and important biochemical enzymes in the testis of streptozotocin-induced diabetic rats. Physiol Pharmacol. (2018) 22:28–37.

[13] Boskabady MH Farkhondeh T. Antiinflammatory, Antioxidant, and immunomodulatory effects of Crocus sativus L. and its Main constituents. Phytother Res. (2016) 30:1072–94. doi: 10.1002/ptr.5622, PMID: 27098287

[14] Bathaie SZ Bolhassani A Tamanoi F. Anticancer effect and molecular targets of saffron carotenoids. Enzyme. (2014) 36:57–86. doi: 10.1016/B978-0-12-802215-3.00004-5, PMID: 27102699

[15] Cerdá-Bernad D Costa L Serra AT Bronze MR Valero-Cases E Pérez-Llamas F . Saffron against neuro-cognitive disorders: an overview of its Main bioactive compounds, their metabolic fate and potential mechanisms of neurological protection. Nutrients. (2022) 14:5368. doi: 10.3390/nu14245368, PMID: 36558528 PMC9781906

[16] Abedimanesh N Ostadrahimi A Bathaie SZ Abedimanesh S Motlagh B Jafarabadi M . Effects of saffron aqueous extract and its main constituent, crocin, on health-related quality of life, depression, and sexual desire in coronary artery disease patients: a double-blind, placebo-controlled, randomized clinical trial. Iran Red Crescent Med J. (2017) 19:e13676. doi: 10.5812/IRCMJ.13676

[17] Zamani M Zarei M Nikbaf-Shandiz M Gholami F Hosseini AM Nadery M . The effects of saffron supplementation on cardiovascular risk factors in adults: a systematic review and dose-response meta-analysis. Front Nutr. (2022) 9:1055517. doi: 10.3389/fnut.2022.1055517, PMID: 36570145 PMC9774508

[18] Samini M Bafandeh F. The protective effects of safranal against diabetes mellitus and its complications. Annu Res Rev Biol. (2017) 15:1–6. doi: 10.9734/ARRB/2017/35478

[19] Zaazaa L Naceiri Mrabti H Ed-Dra A Bendahbia K Hami H Soulaymani A. . Determination of mineral composition and phenolic content and investigation of antioxidant, antidiabetic, and antibacterial activities of Crocus sativus L Aqueous Stigmas Extracts. Adv Pharmacol Pharm Sci. (2021) 2021:7533938. doi: 10.1155/2021/7533938 34195613 PMC8181092

[20] Majidi N Kosari Monfared M Mazaheri-Eftekhar F Movahedi A Karandish M. The effects of saffron petals and damask rose petals on biochemical and inflammatory measurements. J Complement Integr Med. (2021) 19:251–9. doi: 10.1515/jcim-2021-0420 34624188

[21] Pourmasoumi M Hadi A Najafgholizadeh A Kafeshani M Sahebkar A. Clinical evidence on the effects of saffron (Crocus sativus L.) on cardiovascular risk factors: a systematic review meta-analysis. Pharmacol Res. (2019) 139:348–59. doi: 10.1016/j.phrs.2018.11.038, PMID: 30502528

[22] Asbaghi O Soltani S Norouzi N Milajerdi A Choobkar S Asemi Z. The effect of saffron supplementation on blood glucose and lipid profile: a systematic review and meta-analysis of randomized controlled trials. Complement Ther Med. (2019) 47:102158. doi: 10.1016/j.ctim.2019.07.017, PMID: 31779990

[23] Rahmani J Bazmi E Clark C Hashemi Nazari SS. The effect of saffron supplementation on waist circumference, HA1C, and glucose metabolism: a systematic review and meta-analysis of randomized clinical trials. Complement Ther Med. (2020) 49:102298. doi: 10.1016/j.ctim.2020.102298, PMID: 32147057

[24] Giannoulaki P Kotzakioulafi E Chourdakis M Hatzitolios A Didangelos T. Impact of Crocus Sativus L. on metabolic profile in patients with diabetes mellitus or metabolic syndrome: a systematic review. Nutrients. (2020) 12:1424. doi: 10.3390/nu12051424, PMID: 32423173 PMC7284534

[25] Sohaei S Hadi A Karimi E Arab A. Saffron supplementation effects on glycemic indices: a systematic review and meta-analysis of randomized controlled clinical trials. Int J Food Prop. (2020) 23:1386–401. doi: 10.1080/10942912.2020.1807567

[26] Roshanravan B Samarghandian S Ashrafizadeh M Amirabadizadeh A Saeedi F Farkhondeh T. Metabolic impact of saffron and crocin: an updated systematic and meta-analysis of randomized clinical trials. Arch Physiol Biochem. (2022) 128:666–78. doi: 10.1080/13813455.2020.1716020, PMID: 32013614

[27] Moher D Liberati A Tetzlaff J Altman DG. Preferred reporting items for systematic reviews and meta-analyses: the PRISMA statement. J Clin Epidemiol. (2009) 62:1006–12. doi: 10.1016/j.jclinepi.2009.06.005 19631508

[28] Liberati A Altman DG Tetzlaff J Mulrow C Gøtzsche PC Ioannidis JPA . The PRISMA statement for reporting systematic reviews and meta-analyses of studies that evaluate health care interventions: explanation and elaboration. J Clinical Epidemiol. (2009) 62:e1–e34. doi: 10.1016/j.jclinepi.2009.06.006, PMID: 19631507

[29] Higgins JP Altman DG Gøtzsche PC Jüni P Moher D Oxman AD. The Cochrane Collaboration's tool for assessing risk of bias in randomized trials. BMJ. (2011) 343:d5928. doi: 10.1136/bmj.d5928, PMID: 22008217 PMC3196245

[30] Higgins JPT Thomas J Chandler J Cumpston M Li T Page MJ . Cochrane handbook for systematic reviews of interventions version 6.3, Available at: https://www.training.cochrane.org/handbook; (2022), (accessed 1 June 2023)

[31] Higgins JP Thompson SG Deeks JJ Altman DG Measuring inconsistency in meta-analyses. BMJ. (2003) 327:557–60. doi: 10.1136/bmj.327.7414.557, PMID: 12958120 PMC192859

[32] Egger M Davey Smith G Schneider M Minder C. Bias in meta-analysis detected by a simple, graphical test. BMJ. (1997) 315:629–34. doi: 10.1136/bmj.315.7109.629, PMID: 9310563 PMC2127453

[33] Azimi P Ghiasvand R Feizi A Hariri M Abbasi B. Effects of cinnamon, cardamom, saffron, and ginger consumption on markers of glycemic control, lipid profile, oxidative stress, and inflammation in type 2 diabetes patients. Rev Diabet Stud. (2014) 11:258–66. doi: 10.1900/RDS.2014.11.258, PMID: 26177486 PMC5397291

[34] Behrouz V Dastkhosh A Hedayati M Sedaghat M Sharafkhah M Sohrab G. The effect of crocin supplementation on glycemic control, insulin resistance and active AMPK levels in patients with type 2 diabetes: a pilot study. Diabetol Metab Syndr. (2020) 12:59. doi: 10.1186/s13098-020-00568-6, PMID: 32670418 PMC7346493

[35] Ebrahimi F Sahebkar A Aryaeian N Pahlavani N Fallah S Moradi N . Effects of saffron supplementation on inflammation and metabolic responses in type 2 diabetic patients: a randomized, double-blind placebo-controlled trial diabetes. Metab Syndr Obes. (2019) 12:2107–15. doi: 10.2147/DMSO.S216666, PMID: 31686882 PMC6798815

[36] Jaafarinia A Kafami B Sahebnasagh A Saghafi F. Evaluation of therapeutic effects of crocin in attenuating the progression of diabetic nephropathy: a preliminary randomized triple-blind placebo-controlled trial. BMC Complement Med Ther. (2022) 22:262. doi: 10.1186/s12906-022-03744-5, PMID: 36209091 PMC9548209

[37] Karimi-Nazari E Nadjarzadeh A Masoumi R Marzban A Mohajeri SA Ramezani-Jolfaie N . Effect of saffron (Crocus sativus L.) on lipid profile, glycemic indices and antioxidant status among overweight/obese prediabetic individuals: a double-blinded, randomized controlled trial. Clin Nutr ESPEN. (2019) 34:130–6. doi: 10.1016/j.clnesp.2019.07.012, PMID: 31677703

[38] Milajerdi A Jazayeri S Hashemzadeh N Shirzadi E Derakhshan Z Djazayeri A . The effect of saffron (Crocus sativus L.) hydroalcoholic extract on metabolic control in type 2 diabetes mellitus: a triple-blinded randomized clinical trial. J Res Med Sci. (2018) 23:16. doi: 10.4103/jrms.JRMS_286_17 29531568 PMC5842443

[39] Moravej Aleali A Amani R Shahbazian H Namjooyan F Latifi SM Cheraghian B. The effect of hydroalcoholic saffron (Crocus sativus L.) extract on fasting plasma glucose, HbA1c, lipid profile, liver, and renal function tests in patients with type 2 diabetes mellitus: a randomized double-blind clinical trial. Phytother Res. (2019) 33:1648–57. doi: 10.1002/ptr.6351, PMID: 30942510

[40] Sepahi S Mohajeri SA Hosseini SM Khodaverdi E Shoeibi N Namdari M . Effects of Crocin on diabetic maculopathy: a placebo-controlled randomized clinical trial. Am J Ophthalmol. (2018) 190:89–98. doi: 10.1016/j.ajo.2018.03.007, PMID: 29550187

[41] Sepahi S Golfakhrabadi M Bonakdaran S Lotfi H Mohajeri SA. Effect of crocin on diabetic patients: a placebo-controlled, triple-blinded clinical trial. Clin Nutr ESPEN. (2022) 50:255–63. doi: 10.1016/j.clnesp.2022.05.006, PMID: 35871933

[42] Tajaddini A Roshanravan N Mobasseri M Haleem Al-Qaim Z Hadi A Aeinehchi A . The effect of saffron (Crocus sativus L.) on glycemia, lipid profile, and antioxidant status in patients with type-2 diabetes mellitus: a randomized placebo-controlled trial. Phytother Res. (2023) 37:388–98. doi: 10.1002/ptr.7600, PMID: 36580575

[43] Krhač M Lovrenčić MV. Update on biomarkers of glycemic control. World J Diabetes. (2019) 10:1–15. doi: 10.4239/wjd.v10.i1.1, PMID: 30697366 PMC6347654

[44] Chen H Sullivan G Quon MJ. Assessing the predictive accuracy of QUICKI as a surrogate index for insulin sensitivity using a calibration model. Diabetes. (2005) 54:1914–25. doi: 10.2337/diabetes.54.7.1914, PMID: 15983190

[45] Wallace TM Levy JC Matthews DR. Use and abuse of HOMA modeling. Diabetes Care. (2004) 27:1487–95. doi: 10.2337/diacare.27.6.1487 15161807

[46] Hooshmand Moghadam B Rashidlamir A Attarzadeh Hosseini SR Gaeini AA Kaviani M. The effects of saffron (Crocus sativus L.) in conjunction with concurrent training on body composition, glycaemic status, and inflammatory markers in obese men with type 2 diabetes mellitus: a randomized double-blind clinical trial. Br J Clin Pharmacol. (2022) 88:3256–71. doi: 10.1111/bcp.15222, PMID: 35001410

[47] Rajabi A Khajehlandi M Siahkuhian M Akbarnejad A Khoramipour K Suzuki K. Effect of 8 weeks aerobic training and saffron supplementation on inflammation and metabolism in middle-aged obese women with type 2 diabetes mellitus. Sports. (2022) 10:167. doi: 10.3390/sports10110167, PMID: 36355818 PMC9697862

[48] Mohajeri D Mousavi G Doustar Y. Antihyperglycemic and pancreas-protective effects of Crocus sativus L. (saffron) stigma-ethanolic extract on rats with alloxan-induced diabetes. J Biol Sci. (2009) 9:302–10. doi: 10.3923/jbs.2009.302.310

[49] Rajaei Z Hadjzadeh MA Nemati H Hosseini M Ahmadi M Shafiee S. Antihyperglycemic and antioxidant activity of crocin in streptozotocin-induced diabetic rats. J Med Food. (2013) 16:206–10. doi: 10.1089/jmf.2012.2407, PMID: 23437790

[50] Ouahhoud S Lahmass I Bouhrim M Amine K Saalaoui E. Antidiabetic effect of hydroethanolic extract of Crocus sativus stigmas, tepals and leaves in streptozotocin-induced diabetic rats. Physiol Pharmacol. (2019) 23:9–20.

[51] Shirali S Zahra Bathaie S Nakhjavani M. Effect of crocin on the insulin resistance and lipid profile of streptozotocin-induced diabetic rats. Phytother Res. (2013) 27:1042–7. doi: 10.1002/ptr.4836, PMID: 22948795

[52] Dehghan F Hajiaghaalipour F Yusof A Muniandy S Hosseini SA Heydari S . Saffron with resistance exercise improves diabetic parameters through the GLUT4/AMPK pathway in-vitro and in-vivo. Sci Rep. (2016) 6:25139. doi: 10.1038/srep25139, PMID: 27122001 PMC4848502

[53] Mohammad R Daryoush M Ali R Yousef D Mehrdad N. Attenuation of oxidative stress of hepatic tissue by ethanolic extract of saffron (dried stigmas of Crocus sativus L.) in streptozotocin (STZ)-induced diabetic rats. Afr J Pharm Pharmaco. (2011) 5:2166–73. doi: 10.5897/AJPP11.624

[54] Talebanzadeh S Ashrafi M Kazemipour N Erjaee H Nazifi S. Evaluation of the effects of saffron aqueous extract on oxidative stress in the lens of streptozotocin-induced diabetic rats. Biomed Res Ther. (2018) 5:2133–41. doi: 10.15419/bmrat.v5i4.427

[55] Altınöz E Ekici C Özyazgan B Çiğremiş Y. The effects of crocin (active contstituent of saffron) treatment on brain antioxidant enzyme mRNA levels in diabetic rats. Turk J Biochem. (2016) 41:112–7. doi: 10.1515/tjb-2016-0018

[56] Kang C Lee H Jung E-S Seyedian R Jo MN Kim J . Saffron (Crocus sativus L.) increases glucose uptake and in muscle cells via multipathway mechanisms. Food Chem. (2012) 135:2350–8. doi: 10.1016/j.foodchem.2012.06.092, PMID: 22980812

[57] Samarghandian S Azimi-Nezhad M Farkhondeh T. Immunomodulatory and antioxidant effects of saffron aqueous extract (Crocus sativus L.) on streptozotocin-induced diabetes in rats. Indian Heart J. (2017) 69:151–9. doi: 10.1016/j.ihj.2016.09.008, PMID: 28460761 PMC5414951

[58] Samarghandian S Azimi-Nezhad M Farkhondeh T. Crocin attenuate tumor necrosis factor-alpha (TNF-α) and interleukin-6 (IL-6) in streptozotocin-induced diabetic rat aorta. Cytokine. (2016) 88:20–8. doi: 10.1016/j.cyto.2016.08.002, PMID: 27529541

[59] Behrouz V Sohrab G Hedayati M Sedaghat M. Inflammatory markers response to crocin supplementation in patients with type 2 diabetes mellitus: a randomized controlled trial. Phytother Res. (2021) 35:4022–31. doi: 10.1002/ptr.7124, PMID: 33856733

[60] Yaribeygi H Mohammadi MT Rezaee R Sahebkar A. Crocin improves renal function by declining Nox-4, IL-18, and p53 expression levels in an experimental model of diabetic nephropathy. J Cell Biochem. (2018) 119:6080–93. doi: 10.1002/jcb.26806, PMID: 29575259

[61] Motamedrad M Shokouhifar A Hemmati M Moossavi M. The regulatory effect of saffron stigma on the gene expression of the glucose metabolism key enzymes and stress proteins in streptozotocin-induced diabetic rats. Res Pharm Sci. (2019) 14:255–62. doi: 10.4103/1735-5362.258494, PMID: 31160903 PMC6540927

