# Supplementary material for: Effect of saffron supplementation on the glycemic outcomes in diabetes: a systematic review and meta-analysis
Source: Front Nutr. 2024 Mar 15;11:1349006. doi: 10.3389/fnut.2024.1349006 (PMC10978759; doi:10.3389/fnut.2024.1349006)

**Fig. S1: Sensitivity analysis of FPG.**


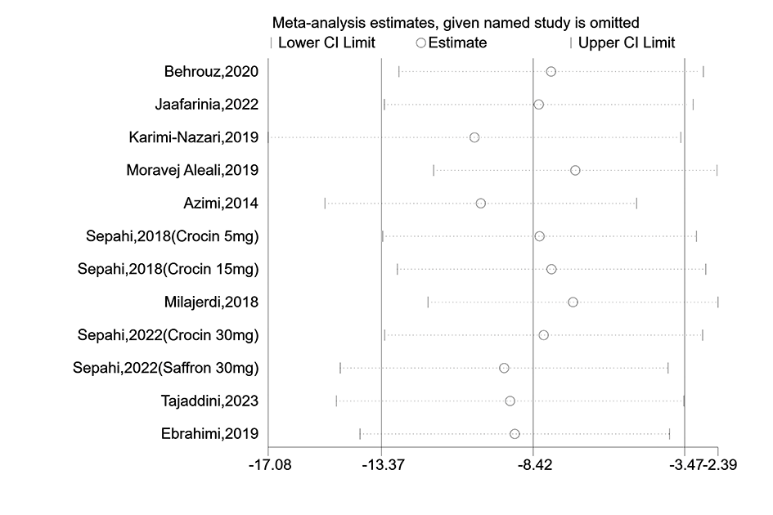


**Fig. S2: Sensitivity analysis of HbA1c.**


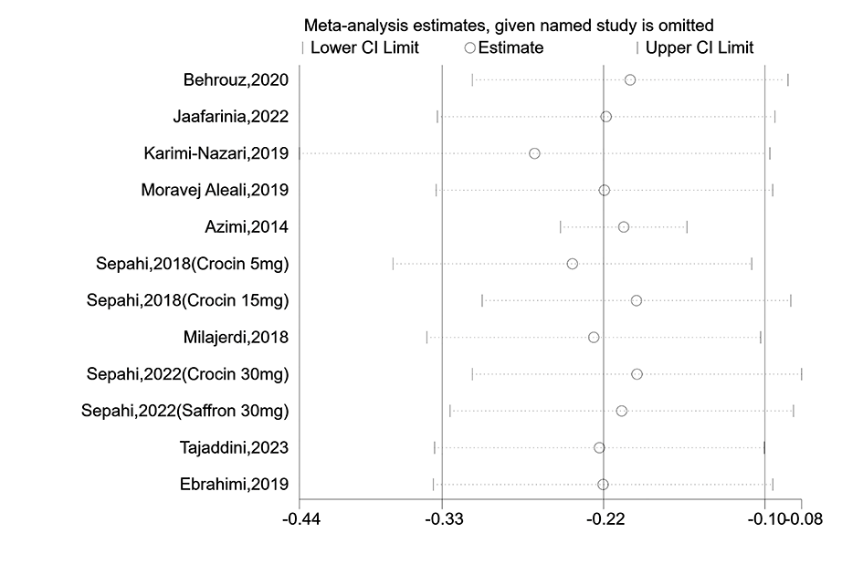


**Fig. S3: Sensitivity analysis of insulin levels.**


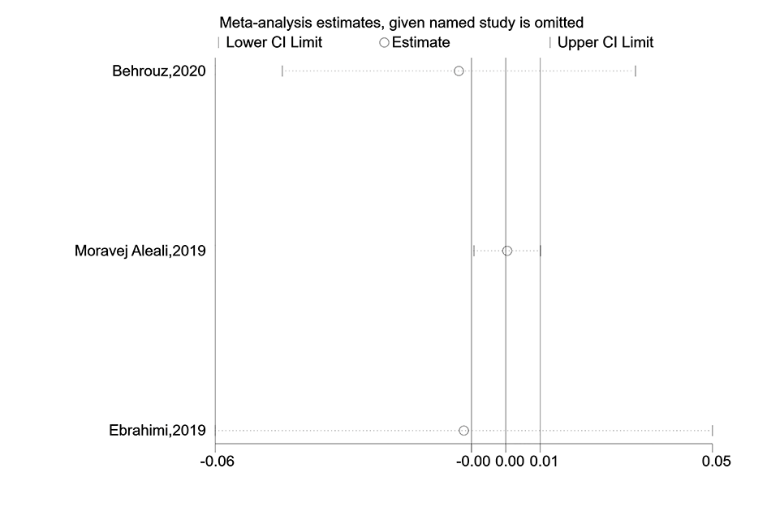


**Fig. S4: Sensitivity analysis of QUICKI.**


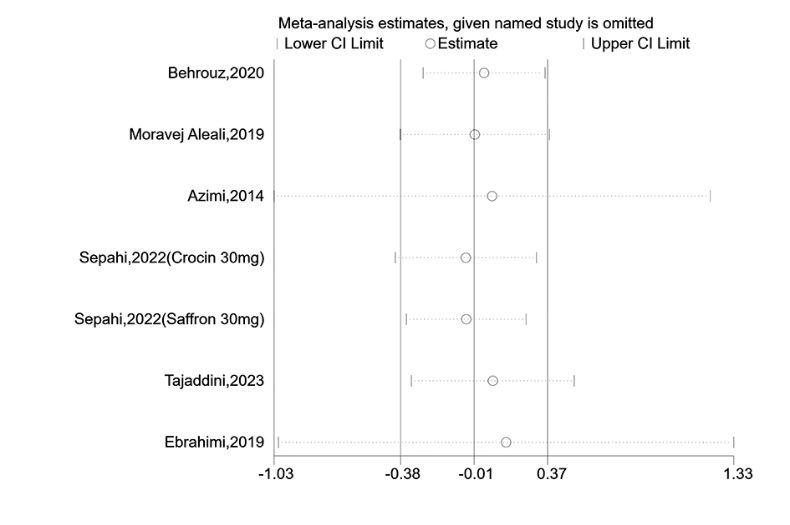


**Fig. S5: Sensitivity analysis of HOMA-IR.**


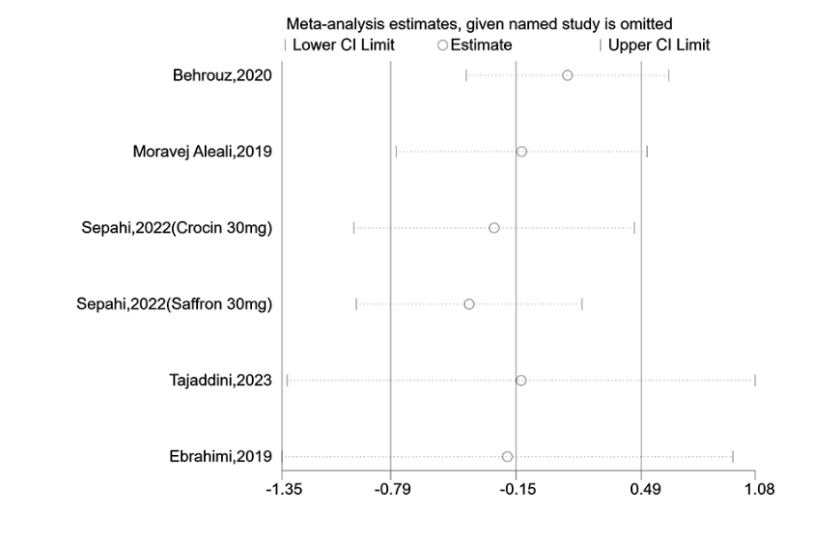


**Fig. S6: Funnel plot of FPG.**


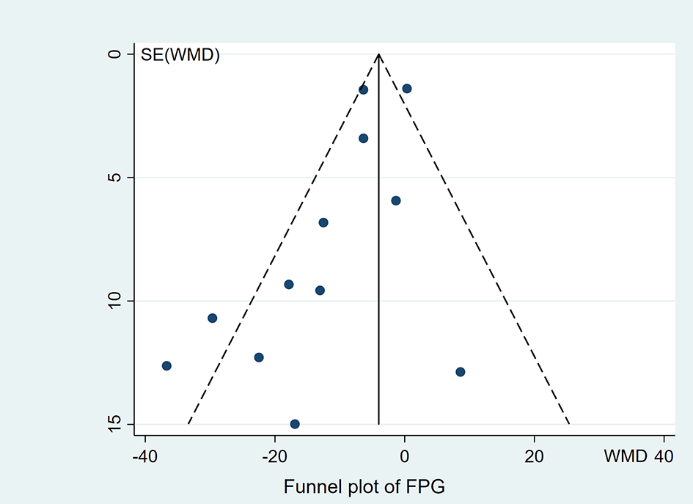


**Fig. S7: Funnel plot of HbA1c.**


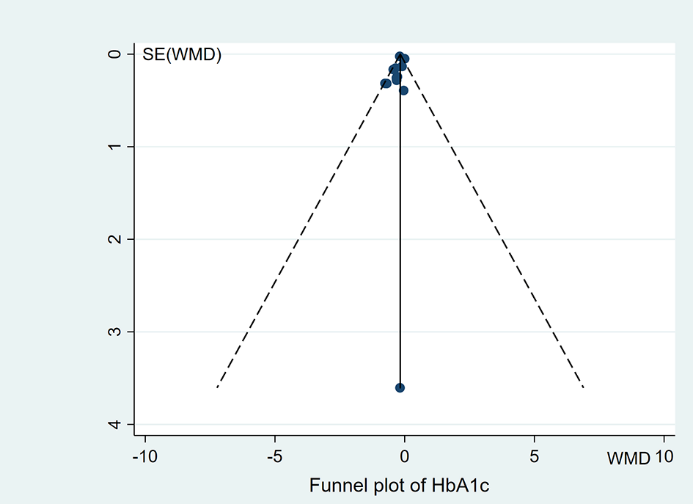

Supplement: Supplementary file 1 [file Data_Sheet_1.docx]
