# Supplementary material for: Effect of saffron supplementation on the glycemic outcomes in diabetes: a systematic review and meta-analysis
Source: Front Nutr. 2024 Mar 15;11:1349006. doi: 10.3389/fnut.2024.1349006 (PMC10978759; doi:10.3389/fnut.2024.1349006)
Supplement: Supplementary file 2 [file Data_Sheet_2.docx]

**Table S1 Search strategy**

| PubMed | Search strategy |
| --- | --- |
| #1 | "Diabetes Mellitus"[MeSH Terms] |
| #2 | "Diabetes Mellitus"[Title/Abstract] OR "Diabetes"[Title/Abstract] OR "Diabetic"[Title/Abstract] OR "DM"[Title/Abstract] |
| #3 | #1 or #2 |
| #4 | "Crocus"[MeSH Terms] |
| #5 | "Crocus"[Title/Abstract] OR "Saffron"[Title/Abstract] OR "Saffrons"[Title/Abstract] OR "Crocus sativus"[Title/Abstract] OR "Saffron Crocus"[Title/Abstract] OR "Crocus, Saffron"[Title/Abstract] |
| #6 | #4 or #5 |
| #7 | #3 and #6 |
| WOS | Search strategy |
| #1 | TS=("Diabetes Mellitus" OR "Diabetes" OR "Diabetic" OR "DM") |
| #2 | TS=("crocus" OR "Saffron" OR "Saffrons" OR "Crocus sativus" OR "Saffron Crocus" OR "Crocus, Saffron") |
| #3 | #1 and #2 |
| Embase | Search strategy |
| #1 | ‘Diabetes Mellitus’/exp |
| #2 | ‘Diabetes Mellitus’:ti,ab,kw OR ‘Diabetes’:ti,ab,kw OR ‘Diabetic’:ti,ab,kw OR ‘DM’:ti,ab,kw |
| #3 | #1 or #2 |
| #4 | ‘Crocus’/exp |
| #5 | ‘Crocus’:ti,ab,kw OR ‘Saffron’:ti,ab,kw OR ‘Saffrons’:ti,ab,kw OR ‘Crocus sativus’:ti,ab,kw |
| #6 | #4 or #5 |
| #7 | #3 and #6 |
| CENTRAL | Search strategy |
| #1 | MeSH descriptor: [Crocus] explode all trees |
| #2 | ("crocus" OR "Saffron" OR "Saffrons" OR "Crocus sativus" OR "Saffron Crocus" OR "Crocus, Saffron"):ti,ab,kw |
| #3 | #1 or #2 |
| #4 | MeSH descriptor: [Diabetes Mellitus] explode all trees |
| #5 | ("Diabetes Mellitus" OR "Diabetes" OR "Diabetic" OR "DM"):ti,ab,kw |
| #6 | #4 or #5 |
| #7 | #3 and #6 |
